# Supplementary material for: CaWRKY30 Positively Regulates Pepper Immunity by Targeting CaWRKY40 against Ralstonia solanacearum Inoculation through Modulating Defense-Related Genes
Source: Int J Mol Sci. 2021 Nov 8;22(21):12091. doi: 10.3390/ijms222112091 (PMC8584995; doi:10.3390/ijms222112091)
Supplement: Supplementary file 1 [file ijms-22-12091-s001.zip › ijms-1412099-supplementary.pdf]

Article

CaWRKY30 Positively Regulates Pepper Immunity by Targeting CaWRKY40 against *Ralstonia solanacearum* Inoculation through Modulating Defense-related Genes

Ansar Hussain[1], Muhammad Ifnan Khan [1], Shahzadi Mahpara [1], Ijaz Rasool Noorka [1], Mohamed A. A. Ahmed [2], Abdullah M. Al-Sadi [3], Bandar S. Aljuaid [4], Ahmed M. El-Shehawi [4], Zhiqin Liu [5,*], Shahid Farooq [6], Ali Tan Kee Zuan [7, *]

[1] Department of Plant Breeding and Genetics, Ghazi University, Dera Ghazi Khan 32200, Pakistan; mifnan@yahoo.com (MIK); aazizwum@gmail.com (SM); sfarooq@gudgk.edu.pk (IRN)

[2] Plant Production Department (Horticulture - Medicinal and Aromatic Plants), Faculty of Agriculture (Saba Basha), Alexandria University, Alexandria 21531, Egypt; mohamed_marie2009@yahoo.com

[3] Department of Plant Sciences, College of Agricultural and Marine sciences, Sultan Qaboos University, Alkhoud, 123, Oman; alsadi@squ.edu.om

[4] Department of Biotechnology, College of Science, Taif University, P.O. Box 11099, Taif 21944, Saudi Arabia, research@bssalj.com (BSA), elshehawi@hotmail.com (AME)

[5] College of Crop Sciences, Fujian Agriculture and Forestry University, Fuzhou, 350001, People's Republic of China

[6] Department of Plant Protection, Faculty of Agriculture, Harran University, Şanlıurfa 63050, Turkey

[7] Department of Land Management, Faculty of Agriculture, Universiti Putra Malaysia, 43400 UPM Serdang, Selangor, Malaysia

* Correspondence: ahtraggar@yahoo.com (AH); tkz@upm.edu.my (AKTZ)

SUPPLEMENTARY MATERIALS

Supplementary Figures

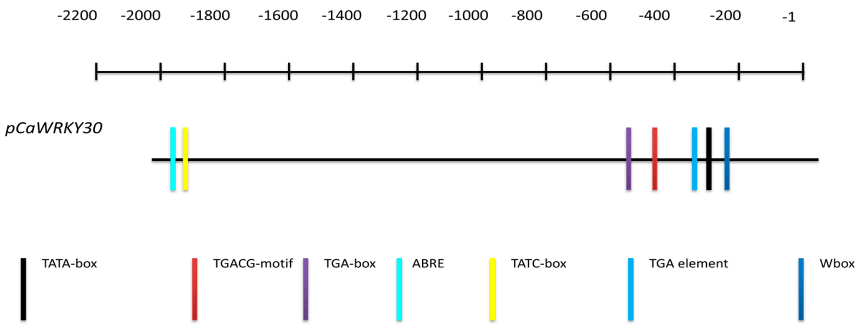

Supplementary Figure S1. Promoter *cis*-elements analysis of *CaWRKY30*

Wbox: transcription factor WRKY binding element; TGA-element: MeJA-relative element; TATC-box: Salicylic responsive; ABRE: Abscisic acid responsive; TGA-box: Auxin responsive; TGACG-motif; TATA-box

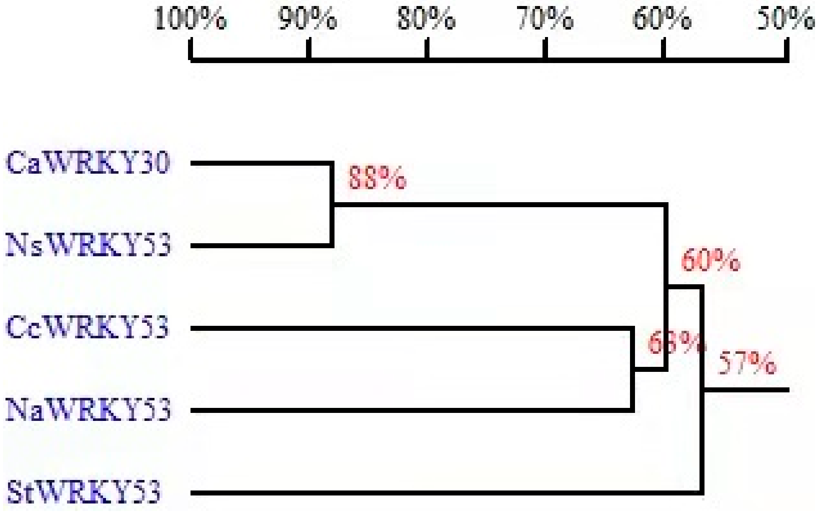

Supplementary Figure S2. Dendrogram presenting amino acid similarity of *CaWRKY30* and WRKYs from other species

CaWRKY30 shares 88%, 60%, 68% and 57% amino acid identities to *NsWRKY53*, *CcWRKY53*, *NaWRKY53* and *StWRKY53*, respectively.

Supplementary Table S1. Pepper-specific primers used for construction of the vectors

Gene	Forward primer (5'-3')	Reverse primer (3'-5')	Size (bP)
CaWRKY30[1]	ATGGATTGTGTTTTTACCTGGGA	TCAGCCCACTTTGACCT	924
CaWRKY30[2]	ATCCGAGAAGCTATTACAG	TCTGAGTCTGAATGGTAC	364
CaWRKY30[3]	ATGGATTGTGTTTTTACCTGGGA	TCAGCCCACTTTGACCT	924

[1]Primers for full length cloning of *CaWRKY30*

[2]Primers for construction of TRV: *CaWRKY30* vector

[3]Primers for construction of 35S:*CaWRKY30-GFP*

Supplementary Table S2. Disease index for *Ralstonia* infected pepper plants

score	Condition level
0	Capsicum plants are normal and asymptomatic at 0.
1	Plants with slight withering, the basal 1-2 leaves withered, the top of pepper plant is normal.
2	Leaves in addition to the top leaves, 1: 2 leaves withered, the top of pepper plant is normal.
3	2/3 of the leaves of pepper plant withered while top pepper plant is normal.
4	Most of the leaves were withered including the top leaves
5	Whole plant was withered or dead.

Supplementary Table S3. Primers used in Real time RT PCR analyses

Gene	Accession number	Forward primers (5'-3')	Reverse primers(3'-5')	Size (bp)
CaWRKY30 [1]	CA01g34480	CACAGGGAGAGGATTTGA	GTAACTTCGCGACAAGT	219
CaPR1	AF348141.1	GCCGTGAAGATGTGGGTCAATGA	TGAGTTACGCCAGACTACCTGAGTA	108
CaNPR1	X61679.1	ACTTCTTCGCCGACGCCAAG	GCCAACACATTCACCAGAGCATC	190
CaDEF1	AF442388	GTGAGGAAGAAGTTTGAAAGAAAGTAC	TGCACAGCACTATCATTGCATACAATTC	267
CaHIR1	AAX20040	CCTGCAATGTTTGCTCATTTGAC	CATGGGAATCGTTGATCTTAATC	164
CaABR1	CA524559	ATGACAGGCACAACAGAAGAAAAT	AATAAGTTATGACAGAGCCATTTT	148
CaActin	GQ339766	AGGGATGGGTCAAAAGGATGC	GAGACAACACCGCCTGAATAGC	225
18srRNA	EF564281	CCGGTCCGCCTATGGTGTGCACCGGTCGTC	GCAGTTGTTCGTCTTTCATAAATCCAAGAA	285
CaWRKY6	KF736800	GGTAGCTAGACAATTATGCTGC	CAAAAAAAAATCTTATCAACTTG	142
CaWRKY22	CA08g07730	GAGGCTGCACAGCTAGTTCCAC	CACCAAGAACAGAGAGGGG	162
CaWRKY27	DQ102364.1	CTGAGCAAGATGATTCCGAGAA	ATTGGCACTGACACCACTCT	148
CaWRKY40	AAX20040.1	AAGTCCAGCAGAGCAGTCAA	AACAATTGTCTAAGCCATCCG	152

[1]**Specific primers to detect relative expression of *CaWRKY30* designed according** to the sequence of 3'UTR

Specific primers for marker genes
